# UVCeed: Leveraging Augmented Reality, Artificial Intelligence, and Gamification for Enhanced Ultraviolet C Disinfection

**DOI:** 10.7759/cureus.80240

**Published:** 2025-03-08

**Authors:** Mitchell K Ng, Michael A Mont, Peter M Bonutti

**Affiliations:** 1 Orthopaedic Surgery, Maimonides Medical Center, Brooklyn, USA; 2 Orthopaedic Surgery, LifeBridge Health, Baltimore, USA; 3 Orthopaedic Surgery, Sarah Bush Lincoln Bonutti Clinic, Effingham, USA

**Keywords:** artificial intelligence, augmented reality, chemical disinfectant, surgical disinfection, ultraviolet c

## Abstract

The growing need for safe and effective surface disinfection solutions has driven innovation beyond traditional chemical disinfectants, which pose challenges including improper application, harmful residues, and environmental concerns. Ultraviolet C (UVC) light has arisen as a chemical-free, reusable alternative, being capable of neutralizing pathogens without leaving residue. However, UVC adoption is hindered by challenges such as ensuring thorough coverage and precise exposure times. This paper introduces the UVCeed Mobile UVC Disinfection Device (Bonutti Technologies, Effingham, Illinois, United States), which leverages augmented reality (AR), artificial intelligence (AI), and gamification to enhance the safety, accuracy, and user experience of UVC disinfection. By providing real-time visual guidance and intuitive feedback, UVCeed addresses the limitations of traditional methods, enabling comprehensive and efficient disinfection. The device's innovative integration of technology positions it as a transformative tool in consumer-grade surface disinfection, combining effectiveness, ease of use, and environmental sustainability with applications in surgery and beyond.

## Introduction and background

Surface disinfection plays a critical role in reducing the spread of infectious diseases, as contaminated surfaces often serve as reservoirs for pathogens such as bacteria, viruses, and fungi [1]. These microorganisms can survive on surfaces for extended periods, creating opportunities for transmission to humans through contact or cross-contamination [2]. The COVID-19 pandemic highlighted the importance of rigorous disinfection practices in decreasing disease spread, particularly in high-touch environments like homes, workplaces, and public spaces [3]. Maintaining clean surfaces is essential not only for infection control but also for ensuring overall public health.

A variety of disinfection methods and devices are currently employed to achieve surface sanitation [4]. Chemical disinfectants, including wipes, sprays, and alcohol-based sanitizers, are commonly used due to their convenience and established effectiveness [5]. However, these products are not without limitations. Improper application, insufficient contact time, or inadequate coverage [3] can render them ineffective [4]. Additionally, chemical disinfectants often leave harmful residues that pose risks to children, pets, and food safety [6]. Repeated exposure to certain chemical agents has also raised concerns about potential health hazards, such as skin irritation, respiratory issues, and even neurological effects [7-9]. Furthermore, the environmental impact of single-use disinfectant products contributes to growing concerns about sustainability [10].

To address the limitations of chemical-based solutions, emerging technologies such as ultraviolet C (UVC) light have gained attention as an alternative disinfection method [11]. UVC light operates within the 200-280 nm wavelength range and is effective at neutralizing pathogens disrupting their deoxyribonucleic acid (DNA) or ribonucleic acid (RNA) makeup [12]. Unlike chemical disinfectants, UVC leaves no residues, is reusable, and is safe for use on food-contact surfaces [13]. Despite its advantages, UVC disinfection comes with challenges, including the invisibility of the light and the need for precise exposure times and thorough coverage to ensure effectiveness [14].

To this end, advanced devices like the UVCeed Mobile UVC Disinfection Device (Bonutti Technologies, Effingham, Illinois, United States) have been introduced to address these issues and represent a significant advancement in surface disinfection technology [15]. By integrating augmented reality (AR), artificial intelligence (AI), and gamification, UVCeed provides users with real-time guidance, intuitive feedback, and visual cues to ensure comprehensive and efficient disinfection [16]. This paper examines the evolution of disinfection technologies, from traditional chemical disinfectants to advanced UVC devices, and introduces UVCeed as a novel solution that bridges the gaps in current practices. By addressing health risks, operational inefficiencies, and environmental concerns associated with traditional methods, UVCeed offers a transformative approach to surface disinfection, setting a new benchmark for efficacy and user accessibility.

## Review

Historical background of UVC technology

The application of ultraviolet (UV) light for disinfection has a long history, evolving over the past century. The germicidal properties of UV light were first observed in the late 19th century when researchers discovered its ability to inactivate bacteria. Early studies identified the shorter wavelengths in the UV spectrum, particularly UVC, as the most effective for sterilization [17].

In the mid-20th century, UVC technology began to be widely used in healthcare settings for air, water, and surface disinfection [17]. UVC lamps were deployed in hospitals to reduce the spread of airborne diseases, and municipal water treatment plants adopted UVC systems to eliminate microbial contamination [18]. By the late 20th century, advancements in UVC lamp design improved the safety and efficiency [19]. The development of compact UVC lamps enabled broader applications, including industrial and commercial use.

In recent decades, UVC technology has become more accessible to consumers. Portable UVC devices such as handheld wands, air purifiers, and sterilization boxes have entered the market, catering to personal and household needs [4]. The COVID-19 pandemic further accelerated the adoption of UVC technology, highlighting its role in mitigating viral transmission [3]. Innovations like the UVCeed Mobile UVC Disinfection Device represent the latest evolution, integrating advanced features such as AR and AI to improve effectiveness and usability [20].

UVCeed: an advancement in UVC technology

The UVCeed Mobile UVC Disinfection Device represents a breakthrough in UVC technology by addressing the common challenges of traditional disinfection methods and enhancing usability through innovative features (Table 1). One of its most significant advantages is the integration of AR, which provides real-time visual feedback, allowing users to see treated and untreated areas on a surface [16]. This feature ensures thorough coverage, eliminating the uncertainty associated with handheld UVC wands. Specifically, UVCeed achieves precise disinfection through camera-based sensors and AI-powered optimization, continuously analyzing key factors such as surface type, distance, angle, and user movement to adjust UV dosage and exposure time [16]. 

**Table 1:** How to use UVCeed: step-by-step guide UVC: ultraviolet C; AR: augmented reality Reference: [15]

Step	What to do	Estimated time
1. Prepare the device	Ensure the UVCeed device is charged and ready. Download and install the UVCeed app if needed.	1-2 minutes (only for initial setup)
2. Pair the device	Turn on the device and connect it to your smartphone via Bluetooth using the app.	30 seconds to 1 minute
3. Select target surface	Identify the surface or object to disinfect. Ensure it is clear of obstructions or reflective materials.	Immediate
4. Position the device	Hold the UVCeed device 1-4 inches above the surface and activate it through the app or device.	Immediate
5. Follow AR guidance	Use the AR overlay on your smartphone to monitor treated and untreated areas in real time. Move steadily across the surface.	
	Small objects (e.g., phones, keys, etc.): 15-30 seconds per side.	
	Medium surfaces (e.g., desks, countertops, etc.): 1-2 minutes.	
	Large surfaces (e.g., tables, floors, etc.): 3-5 minutes or more.	
6. Maintain dwell time	Keep the device at a consistent distance and follow app prompts for sufficient exposure time.	Automatically adjusted by the app
7. Pause for safety	If a human or pet is detected nearby, the device will pause UVC output. Wait until the area is clear to resume.	Variable, depending on the situation
8. Complete disinfection	Continue until the app confirms all areas are disinfected. The AR overlay will show completion.	Varies by surface size (see step 5)
9. Review session summary	Review the app's summary to confirm coverage and disinfection details.	10-30 seconds
10. Power off and store	Turn off the device and store it in a safe place. Recharge if needed for future use.	Immediate

The incorporation of AI further elevates its functionality by optimizing UVC dosage/exposure times based on factors including surface type, distance, and user movement, ensuring effective pathogen inactivation [16]. UVCeed also incorporates gamification elements, such as visualized pathogen reduction, which both engage users and encourage adherence to proper disinfection practices. Safety is another significant advantage, as the device uses machine vision to detect the presence of humans or pets, disabling UVC output to prevent accidental exposure automatically [18]. Its compact and portable design makes it versatile for use on a variety of surfaces, from household items to high-touch public spaces, while its intuitive smartphone-based interface ensures accessibility for users of all experience levels. Together, these features position UVCeed as a user-friendly, safe, and highly efficacious tool for surface disinfection.

Uses and applications of UVC technology

UVC technology is versatile, with applications spanning various industries [4,12]. In healthcare, UVC is used for disinfecting surgical tools, hospital rooms, and high-risk areas to prevent healthcare-associated infections. Water treatment facilities utilize UVC systems to purify drinking water and treat wastewater by inactivating harmful microorganisms without chemicals [21]. In the food industry, UVC light is employed to sanitize surfaces, packaging, and even food products, enhancing safety and shelf life. In residential and commercial spaces, UVC devices help maintain hygiene by disinfecting surfaces and improving air quality. The technology is also used in transportation systems, such as airplanes and public transit, to sanitize high-touch areas.

UVC technology holds significant potential in the field of surgery (e.g., general surgery, neurosurgery, cardiothoracic surgery), particularly in orthopedic surgery, where maintaining sterility is critical to patient outcomes [2]. In surgical environments, UVC light can be used to disinfect operating rooms, surgical instruments, and air circulation systems, ensuring a sterile environment that minimizes the risk of postoperative infections [22]. Orthopedic surgeries, which often involve implants and prosthetics, are especially vulnerable to infection due to the introduction of foreign materials. UVC technology can play a vital role in sterilizing not only the surgical instruments but also the implant surfaces, reducing the risk of microbial contamination [19]. Furthermore, portable UVC devices could be used intraoperatively to disinfect surgical fields or surrounding areas in real time, adding an extra layer of protection. The application of UVC in air sterilization systems within operating rooms also helps mitigate airborne microbial load, creating a safer surgical environment [21]. These advancements could significantly enhance infection control protocols in orthopedic surgery, where the prevention of infections such as osteomyelitis or periprosthetic joint infections is paramount for successful patient outcomes.

Competing UVC technologies

Handheld Wands

Traditional handheld UVC wands are portable and affordable, making them a popular choice for consumers. However, these devices lack feedback mechanisms, requiring users to estimate coverage and exposure time, which often leads to inconsistent results [23].

Static UVC Units

Static UVC units are effective for disinfecting small items like phones and keys. While they provide controlled disinfection in enclosed spaces, they are limited in application and unsuitable for larger or irregular surfaces.

UVC Air Purifiers

UVC air purifiers combine UVC technology with filtration systems to neutralize airborne pathogens. These devices are effective for improving indoor air quality but are not designed for surface disinfection [24].

Autonomous UVC Robots

Autonomous UVC robots are used in healthcare and industrial settings to disinfect large areas. They provide comprehensive coverage but are expensive, bulky, and impractical for personal use [25].

Role of UVC in surgery, specifically orthopedic surgery

In surgical settings, maintaining a sterile environment is critical to reducing the risk of infections, especially in procedures that involve implants and prosthetics [26]. Orthopedic surgery, which frequently involves the use of foreign materials like plates, screws, and/or joint arthroplasty, is uniquely susceptible to infections such as osteomyelitis and periprosthetic joint infections [26]. UVC technology can play an important role in enhancing sterility in these environments. UVC can be used to disinfect surgical tools, implants, and the surrounding environment, ensuring minimal microbial contamination. It can also be applied intraoperatively to sterilize surfaces and surgical fields in real time, providing an additional layer of protection [26]. Furthermore, UVC devices integrated into air sterilization systems help reduce airborne microbial load in operating rooms, creating a safer environment for both patients and surgical teams. The use of portable UVC devices like UVCeed further enhances infection control, allowing precise disinfection of irregularly shaped surgical instruments and hard-to-reach areas, ensuring appropriate operative sterility [15,23].

Table 2 highlights UVCeed's technological advancements, demonstrating its most notable advantages over traditional UVC devices and other competitors. While it has limitations such as cost and dependence on a smartphone interface (average cost is ~$130), UVCeed stands out for its effectiveness, safety, and versatility, making it a leading option in consumer-grade UVC technology.

**Table 2:** Comparative Analysis: UVCeed vs. chemical and UVC alternatives UVC: ultraviolet C; AI: artificial intelligence; AR: augmented reality Reference: [20]

Aspect	UVCeed	Chemical disinfectants	Traditional UVC devices
Effectiveness	AI ensures consistent pathogen inactivation.	Dependent on proper application; prone to misuse.	Inconsistent results due to lack of feedback mechanisms.
User safety	Machine vision prevents accidental exposure.	Risk of skin irritation, respiratory issues, and residue.	Limited safety features; risk of UVC overexposure.
Environmental impact	Chemical-free and reusable, reducing ecological footprint.	Generates waste; contains potentially hazardous compounds.	Similar environmental benefits but less precise application.
Ease of use	AR-guided feedback ensures thorough coverage.	Requires precise timing and technique for effectiveness.	Relies on user judgment, increasing the likelihood of errors.
Cost and accessibility	Higher upfront cost, but cost-effective over time due to reusability.	Initially cheaper but less sustainable.	Varies; basic devices are cheaper but less feature-rich.

Future uses and areas of research for UVCeed

The potential applications of UVCeed extend far beyond its current uses, with technological advancements and ongoing research likely to unlock new opportunities for disinfection practices. In healthcare, UVCeed could be further integrated into specialized medical settings, such as intensive care units, neonatal wards, and surgical environments [24,25]. These spaces require a high degree of precision in disinfection, and UVCeed's AI-driven optimization and AR-guided feedback make it an ideal candidate for ensuring comprehensive sterilization. By integrating UVCeed with hospital networks and IoT systems, real-time monitoring of sterilization practices could become standard, enabling better compliance with hygiene protocols and identifying areas for improvement [27].

Another promising avenue for UVCeed is its integration with robotic platforms. By combining its AR and AI capabilities with autonomous navigation systems, UVCeed could be adapted for hands-free disinfection of large or complex environments such as airports, hospitals, or sports arenas. This would automate the process of calculating coverage and ensure thorough application in high-traffic spaces, reducing human effort while enhancing precision. Its portability and efficacy also position UVCeed as an essential tool in emergency and disaster response scenarios, where rapid and effective disinfection is critical [23].

The transportation sector represents another significant opportunity for UVCeed. As public health concerns continue to shape transportation protocols, UVCeed could be adapted for disinfection in buses, trains, airplanes, and other public transit systems [18]. Its ability to disinfect seats, handrails, and air circulation systems would enhance safety for travelers while minimizing downtime between uses [18]. In the consumer market, miniaturized versions could be developed for wearable applications, allowing individuals to disinfect personal items like phones, masks, and clothing, providing an additional layer of protection for people in high-risk environments or for frequent travelers [19].

Research into UVCeed's capabilities is likely to expand its functionality and refine its design. Efforts to optimize UVC delivery mechanisms for irregular surfaces, complex geometries, and sensitive materials could make the device more versatile. Investigating the long-term effects of UVC exposure on different materials may lead to innovations such as UVC-resistant coatings, which would protect equipment and infrastructure in environments where frequent disinfection is required [23]. Another area of interest is pathogen-specific customization, where AI algorithms could dynamically adjust UVC dosage based on the type of microorganism being targeted. This would allow UVCeed to combat resistant strains or emerging pathogens more effectively [19].

User behavior and engagement also represent a crucial focus for future research. Understanding how gamification elements influence adherence to disinfection protocols could help refine UVCeed's interactive features. By studying how users interact with the device over time, developers could make interfaces more intuitive and adapt feedback systems to meet the needs of diverse populations [18]. These advancements would not only improve the user experience but also ensure that UVCeed remains a trusted tool for achieving effective disinfection. Compared to chemical disinfectants, UVCeed offers several distinct advantages. While chemical disinfectants are widely available and inexpensive, they often leave harmful residues and require meticulous application to ensure effectiveness [18]. UVCeed addresses these issues by providing a residue-free, reusable solution that minimizes health risks associated with chemical exposure [24,27]. The higher upfront cost of UVCeed may deter some users, though its durability and lack of consumable supplies make it cost-effective over time.

When compared to traditional UVC devices, UVCeed's integration of AR and AI sets it apart. Conventional UVC wands and static units often rely on user judgment to determine coverage, which increases the likelihood of incomplete disinfection [27]. UVCeed's real-time visual feedback ensures consistent and thorough application, reducing the risk of error. Its gamification features further enhance its appeal by engaging users and encouraging adherence to best practices. In contrast, UVC robots, while highly effective for large-scale disinfection, are expensive and impractical for individual or household use [24]. UVCeed fills this gap by offering comparable precision in a portable and affordable format, making it accessible to a broader audience.

UVCeed represents a critical advancement in disinfection technology, combining precision, safety, and versatility in a single device [26]. Its future applications in healthcare, transportation, disaster response, and wearable technology highlight its potential to redefine hygiene standards across industries [28]. Ongoing research into its optimization and adaptability will only enhance its effectiveness, ensuring that it remains at the forefront of UVC disinfection solutions. Compared to both chemical and traditional UVC alternatives, UVCeed's innovative features make it a transformative tool for creating safer, cleaner environments worldwide.

## Conclusions

UVCeed represents a significant advancement in the field of disinfection, combining cutting-edge technologies to address the limitations of traditional methods. Its potential future applications, ranging from healthcare to wearable devices, highlight its versatility and adaptability. Research focused on enhancing delivery mechanisms, expanding compatibility, and optimizing pathogen-specific responses will further solidify its position as a leader in UVC technology. Compared to chemical disinfectants and traditional UVC devices, UVCeed offers superior safety, precision, and environmental benefits. While its upfront cost may pose challenges, its long-term value and advanced features make it a transformative tool in public health and hygiene. As UVC technology continues to evolve, UVCeed's integration into broader disinfection strategies will play a critical role in promoting safer, cleaner environments worldwide.
